# Prokaryotic Soluble Overexpression and Purification of Bioactive Human Growth Hormone by Fusion to Thioredoxin, Maltose Binding Protein, and Protein Disulfide Isomerase

**DOI:** 10.1371/journal.pone.0089038

**Published:** 2014-03-10

**Authors:** Minh Tan Nguyen, Bon-Kyung Koo, Thu Trang Thi Vu, Jung-A Song, Seon-Ha Chong, Boram Jeong, Han-Bong Ryu, Sang-Hyun Moh, Han Choe

**Affiliations:** 1 Department of Physiology and Bio-Medical Institute of Technology, University of Ulsan College of Medicine, Seoul, Korea; 2 Bio-FD&C Co. Ltd., Incheon, Korea; St. Georges University of London, United Kingdom

## Abstract

Human growth hormone (hGH) is synthesized by somatotroph cells of the anterior pituitary gland and induces cell proliferation and growth. This protein has been approved for the treatment of various conditions, including hGH deficiency, chronic renal failure, and Turner syndrome. Efficient production of hGH in *Escherichia coli* (*E. coli*) has proven difficult because the *E. coli*-expressed hormone tends to aggregate and form inclusion bodies, resulting in poor solubility. In this study, seven N-terminal fusion partners, hexahistidine (His6), thioredoxin (Trx), glutathione S-transferase (GST), maltose-binding protein (MBP), N-utilization substance protein A (NusA), protein disulfide bond isomerase (PDI), and the b′a′ domain of PDI (PDIb′a′), were tested for soluble overexpression of codon-optimized hGH in *E. coli*. We found that MBP and hPDI tags significantly increased the solubility of the hormone. In addition, lowering the expression temperature to 18°C also dramatically increased the solubility of all the fusion proteins. We purified hGH from MBP-, PDIb′a′-, or Trx-tagged hGH expressed at 18°C in *E. coli* using simple chromatographic techniques and compared the final purity, yield, and activity of hGH to assess the impact of each partner protein. Purified hGH was highly pure on silver-stained gel and contained very low levels of endotoxin. On average, ∼37 mg, ∼12 mg, and ∼7 mg of hGH were obtained from 500 mL-cell cultures of Trx-hGH, MBP-hGH, and PDIb′a′-hGH, respectively. Subsequently, hGH was analyzed using mass spectroscopy to confirm the presence of two intra-molecular disulfide bonds. The bioactivity of purified hGHs was demonstrated using Nb2-11 cell.

## Introduction

Human growth hormone (hGH), also known as somatotropin or somatropin, contains 191 amino acid residues and plays important roles in growth control, promotion of growth and development of cells, and regulation of many metabolic processes. GH is secreted by the pituitary gland and circulates in the bloodstream, where it binds to the cell surface GH receptor (GHR) and activates the GHR/JAK2 (Janus kinase 2) complex [1]. Physiologically, hGH maintains positive nitrogen balance and triggers protein synthesis in muscle cells [2], increases the amino acid uptake into skeletal muscle [3], as well as regulates longitudinal bone growth [4]. Moreover, hGH protects cardiac myocytes and lymphoid cells against apoptosis [5], [6]. Since the 1960s, hGH has been used in the regulation of normal growth in GH-deficient children [7]. Due to its variety of biological functions, hGH has been approved in a wide range of therapeutic treatments such as for adult GH deficiency, chronic renal failure, Turner syndrome, and HIV infection [8]–[11]. hGH derived from the pituitary is scarce, thus initially limiting its use in therapeutic treatments. With the development of recombinant DNA technology, the hGH gene was cloned in 1979 and recombinant hGH was approved for clinical use in 1985 [7], [12].

hGH is mainly produced in eukaryotic expression systems, such as in yeast, larva, or mammalian cells [13]–[16]. However, these expression systems have several limitations: the unexpected hyper-glycosylation in yeast resulting in high immunogenicity; and the requirement of specialized equipment for large-scale production and high cost in mammalian systems. Prokaryotic expression systems such as *E. coli* are widely used for mass production of recombinant proteins. However, overexpression of protein in *E. coli* results in inclusion bodies due to misfolding and aggregation of the protein [17]–[21]. In general, inclusion bodies need to be solubilized using high concentrations of denaturants such as urea or guanidine hydrochloride, and refolded by the removal of denaturants. In many cases, however, the overall yield of biologically active protein from inclusion bodies is low [22]. To improve the solubility of hGH in *E. coli*, several strategies have been attempted, including periplasmic secretion [23], [24], low expression temperature [25], and low isopropyl-β-D-thiogalactoside (IPTG) concentration [26]. These methods all successfully improve the solubility of hGH. However, even with these methods, the final yield of secreted pelB- and ompA-hGH into periplasmic only approximately 1.4 mg/L. In addition, the study of slowing the protein synthesis rate by regulating the IPTG concentration did not provide detailed information about purification [26]. During the preparation of this manuscript, maltose-binding protein (MBP) was reported as a fusion partner for soluble expression of tandem dimer of thrombopoietin mimetic peptide (dTMP) fused to hGH (dTMP-hGH) [27].

In this study, seven N-terminal fusion tags, hexahistidine (His6), thioredoxin (Trx), glutathione S-transferase (GST), MBP, N-utilization substance protein A (NusA), protein disulfide bond isomerase (PDI), and the b′a′ domain of PDI (PDIb′a′), were tested for the soluble overexpression of hGH in *E. coli*. Among these seven tags, MBP and PDI significantly increased the solubility at 37°C. Lowering the expression temperature to 18°C dramatically increased the solubility of all fusion proteins. We then purified hGHs from Trx-hGH, MBP-hGH, and PDIb′a′-hGH using conventional chromatographic techniques and compared the final purity, yield, and activity to understand the effects of these tags. Purified hGH was analyzed by mass spectroscopy, and its bioactivity was confirmed using Nb2-11 cells.

## Materials and Methods

### Plasmid construction and bacterial expression of expression vectors

The DNA sequence encoding 191 amino acids of mature hGH (NCBI Reference Sequence: AAA98618.1) was codon-optimized for *E. coli* expression and synthesized (Epoch life science, Missouri, TX). The tobacco etch virus recognition site (TEVrs), ENLYFQ/G, was placed at the N-terminus of hGH. Two adapter sequences, attB1 site (5′-GGGGACAAGTTTGTACAAAAAAGCAGGCTTC-3′) and attB2 site (5′-ACCCAGCTTTCTTGTACAAAGTGGTCCCC-3′), were also attached at the 5′-end and 3′-end of the DNA sequence, respectively. Synthesized DNA was sub-cloned into pDONOR207 by BP recombination reaction [28], resulting in entry clone pENTR-hGH. Using LR recombination reaction [28], the pENTR-hGH plasmid was recombined with the destination vectors, pDEST-HGWA, pDEST-HXGWA, pDEST-HGGWA, pDEST-HMGWA, pDEST-HNGWA, pDEST-PDI, and pDEST-PDIb′a′, which have either a His6 or His8 at the N-terminus of each tag sequence [29], [30]. The sequences of all expression clones were confirmed with sequencing (Macrogen, Daejeon, Korea).

The seven expression plasmids were transformed into *E. coli* BL21(DE3) cells and cultured in LB broth at 37°C at 200 rpm. A single colony from plated transformants was inoculated into 4 mL of LB medium containing 50 µg/mL ampicillin, grown at 37°C overnight, and then transferred to LB medium including 50 µg/mL ampicillin at a ratio of 1∶200. The culture was grown at 37°C in a shaking incubator at 200 rpm. Once the OD_600_ reached 0.6, 0.5 mM IPTG was added to the culture medium to induce protein expression with the temperature maintained at 37°C for 4 h or decreased to 18°C for 18 h. After cultivation, the protein expression level was analyzed by SDS-PAGE using a 10% tricine gel.

### Purification of hGH from Trx-hGH

The 500 mL cell culture was collected by centrifugation at 3,800× g for 30 min. Cell pellets were then resuspended in buffer A1 (20 mM Tris-HCl, pH 8.0, 5% glycerol (v/v), 0.2% TritonX-100 (v/v)) supplemented with 0.2% protease inhibitor cocktail (v/v; Sigma-Aldrich, St. Louis, MO) at a concentration of 50 mL/g. The ultrasonic cell disruptor JY99-IIDN (Ningbo Scientz Biotechnology, Guangdong, China) was used to homogenize the cells on ice at 1,000 W for 40 cycles of 10 seconds, followed by intervals of 50 seconds for cooling. Cell debris from the lysate was removed by centrifugation at 23,000× g for 30 min, and the supernatant fraction was filtered with 0.2 µm Acrodisc Syringe Filters, Supor Membrane (Pall Corporation, Ann Arbor, MI). Trx-hGH protein from the 500 mL cell supernatant was used as a crude sample for the first purification by immobilized metal ion affinity chromatography (IMAC). The supernatant containing Trx-hGH was loaded onto a 20 mL HisPrep FF 16/10 column (GE Healthcare, Piscataway, NJ) pre-equilibrated with buffer A1. The column was adequately washed with 10–15 column volume (CV) of buffer A1 and 10–15 CV of buffer A2 (20 mM Tris-HCl, pH 8.0, 5% glycerol (v/v)). Subsequently, protein samples were eluted with 5 CV of 10% buffer B1 (20 mM Tris-HCl, pH 8.0, 5% glycerol (v/v), 1 M imidazole). The partially purified TrX-hGH protein was diluted two times with buffer A2 and then treated with purified TEV protease at a ratio of 1∶10 (w/w) for proteolytic cleavage with the addition of 1 mM dithiothreitol (DTT). The reaction was left to set at 4°C for 4 h. NaCl was then added to the cleaved sample at a final concentration of 500 mM with or without 2.5 mM of DTT, and the sample was loaded again onto IMAC equilibrated with buffer A3 (20 mM Tris-HCl, pH 8.0, 500 mM NaCl, 5% glycerol (v/v)) with or without 2.5 mM DTT. Most of the cleaved hGH protein passed through the column and was collected for the next purification step. Column-bound TEV protease and Trx tag separated from hGH were eluted with 5 CV of 100% buffer B2 (20 mM Tris-HCl, pH 8.0, 500 mM NaCl, 5% glycerol (v/v), 1 M imidazole). To obtain the pure monomer form of hGH, the sample was pooled and injected into the column packed with 500 mL of Superdex 200 prep grade (GE Healthcare) pre-equilibrated with buffer C (20 mM Tris-HCl, pH 8.0, 200 mM NaCl, 10 mM EDTA, 5% glycerol (v/v)) with or without 2.5 mM DTT and 0.2% protease inhibitor cocktail (v/v) (Sigma-Aldrich). Fractions of pure hGH were dialyzed against PBS buffer and lyophilized for further experiments. All fractions during all purification process were analyzed by SDS-PAGE using 10% tricine SDS-PAGE gel. The concentrations of proteins were determined by the Bradford method using BSA as the standard [31].

### Purification of hGH from MBP-hGH and PDIb′a′-hGH

The cell mass was harvested from culture broth by centrifugation at 3,500 rpm for 30 min and resuspended in buffer A3 containing 1 mM phenylmethylsulfonylfluoride (PMSF) at a concentration of 20 mL/g. The supernatant fraction containing MBP-hGH or PDIb′a′-hGH was also obtained using the procedure described above. Similar to Trx-hGH, the MBP-hGH or PDIb′a′-hGH protein from 500 mL cell culture was initially purified with IMAC. Filtered supernatant was loaded to a 20 mL HisPrep FF 16/10 column (GE Healthcare) pre-equilibrated with buffer A3. In order to remove non-specifically bound proteins, the column was adequately washed with 10–15 CV of 5% buffer B2, and then protein samples were eluted with 5 CV of 100% buffer B2. The partially purified MBP-hGH or PDIb′a′-hGH protein was dialyzed against TEV standard buffer (50 mM Tris-HCl, pH 8.0, 0.5 mM EDTA, 1 mM DTT, 5% glycerol (v/v)) before proteolytic cleavage with purified TEV protease at 18°C with a ratio of 1∶30 (w/w). After 2 h, the cleaved sample was pooled and dialyzed against buffer A3 to effectively remove EDTA and DTT before it was applied again onto a 20 mL HisPrep FF 16/10 column. The column was washed with 5 CV of buffer A3, and then the cleaved hGH was eluted with 5 CV of 5% buffer B2. Finally, the sample was pooled and injected onto a HiLoad 16/600 Superdex-75 column (GE Healthcare) pre-equilibrated with buffer C. Fractions containing pure hGH were dialyzed against PBS buffer and lyophilized for further experiments. All fractions during all purification steps were analyzed by SDS-PAGE using 10% tricine SDS-PAGE gel. The concentration of proteins was determined by the Bradford method using BSA as the standard.

### Purification of TEV protease

The plasmid pRK793 encoding for the TEV protease mutant protein [32] was transformed in BL21(DE3) cells to express the protease as a soluble form. The same methods for cell culture and induction were followed as described above except that the induction conditions were set at 30°C for 6 h. The supernatant preparation of TEV protease was performed following a similar method of MBP-hGH as described above. The cell pellet was harvested and resuspended in buffer A3 containing 1 mM PMSF at a concentration of 10 mL/g. The cell suspension was sonicated on ice at 1,500 W for 20–30 cycles of 10 seconds followed by intervals of 40 seconds for cooling. The supernatant was loaded onto a 20 mL HisPrep FF 16/10 column (GE Healthcare) pre-equilibrated with buffer A3 for binding the TEV protease to the column. A 10 CV of 10% buffer B2 was used for the washing step to remove nonspecifically bound proteins. The TEV protease was eluted with 5 CV of 100% buffer B2, and 4 mM DTT was added to the eluate to prevent precipitation. The protein was pooled and concentrated to a volume of 10–15 mL using an Amicon Ultra-15 Centrifugal Filter Units (Millipore, Billerica, MA) and then loaded onto a HiPrep 16/10 Desalting column (GE Healthcare) to remove salt and change to stock buffer (50 mM Tris-HCl, pH 8.0, 1 mM EDTA, 0.1% Triton X-100 (v/v), 10% glycerol (v/v)). Subsequently, an additional 5 mM of DTT and 50% glycerol (v/v) were added to the TEV protease for storing. The final product was aliquoted and stored at −80°C, and the activity of the purified TEV protease was 7 units/µL (1 unit of the purified TEV protease cleaves ≥85% of 2 µg of the tag fused protein overnight at 18°C).

### MALDI-TOF MS analysis

For sample preparation, 5 µg of protein in each sample was treated with or without 10 mM DTT to yield reduced and non-reduced samples, respectively. After 10 min of DTT treatment on ice, samples were precipitated with 10% trichloroacetic acid (v/v; Sigma-Aldrich) and collected by centrifugation at 12,000 rpm for 10 min. The pellets were resuspended in buffer consisting 0.5 M Tris-HCl, pH 8.0, 5% Glycerol (v/v), 100 mM NaCl, 1 mM EDTA, 2% SDS, and 50 mM iodoacetamide (IAA). The reaction was left to set at room temperature for 1 h in the dark, and protein samples were then loaded onto a 10% tricine SDS-PAGE gel. Gel fragments of the target protein were cut out and incubated in a buffer containing 200 mM ammonium hydrogen carbonate and 40% acetonitrile (ACN, v/v) at 37°C for 10 min; this process was repeated until the gel was completely destained. Gels were then dried by a vacuum concentrator (Eppendorf, Hamburg, Germany) and treated with 10 µL of buffer containing 20 µg/mL trypsin, 40 mM ammonium hydrogen carbonate, 9% ACN (v/v), and 1 mM HCl. After overnight incubation at 37°C, gel fragments were removed and the supernatant was spotted on the plate for MALDI-TOF MS at a 1∶1 ratio with 10–20 mg/mL matrix α-Cyano-4-hydroxycinnamic acid (CHCA, Sigma-Aldrich) dissolved in buffer containing 70% ACN (v/v) and 0.1% trifluoroacetic acid (v/v). The analysis was performed using Voyager-DE STR (Applied Biosystems, South San Francisco, CA). Mass data were analyzed by the Data Explorer software (Applied Biosystems, South San Francisco, CA). The obtained peptide mass results were then searched against the NCBI database using the Mascot peptide mass fingerprinting search program (Matrix Science, Boston, MA) [33].

### Electrophoresis and silver staining

Protein fractions were treated with 5× sample buffer (312.5 mM Tris-HCl, pH 6.8, 50% glycerol, 5% SDS, 0.05% bromophenol blue with or without 100 mM DTT) to prepare the reduced or non-reduced samples, respectively, for SDS-PAGE analysis. Reduced samples were loaded onto a 10% tricine SDS-PAGE gel for separation of protein bands stained with Coomassie brilliant blue R-250 (Amresco, Solon, OH) afterwards. The expression and solubility levels were evaluated with ImageJ image analysis software (http://imagej.nih.gov/ij) by measuring the percentage of expressed fusion protein over the total protein amount synthesized by *E. coli* and the percentage of the soluble fraction over the amount of total expressed fusion protein, respectively.

For purity qualification, gels consisting of reduced and non-reduced samples were silver-stained using the Silver Stain Plus kit (Bio-Rad Laboratories, Hercules, CA). The gels were fixed and rinsed with mild agitation for 20 min in Fixative Enhancer Solution and distilled water, respectively. To visualize the protein bands, the gel was placed in staining solution for approximately 20 min. The developed gel was transferred to a solution of 5% acetic acid to stop the reaction.

### Endotoxin assay of hGH

Endotoxins in the final purified hGH protein were removed by a previously described method [34]. Briefly, the QCL-1000 Endpoint Chromogenic Limulus Amebocyte Lysate (LAL) Assay kit (Lonza, Basel, Switzerland) was used to measure the endotoxin level of purified hGH. Samples of 50 µL of hGHs, standards, and endotoxin-free water were dispensed onto a 96 well-plate pre-equilibrated at 37°C followed by the addition of 50 µL of LAL to each well. After 10 min, 100 µL of substrate solution prewarmed to 37°C was added to the samples. The samples were pipetted consistently and incubated for 16 min. Then, 100 µL of stop reagent (25% v/v glacial acetic acid in water) was added to stop the reaction. The absorbance of each sample at 405–410 nm was measured using a spectrophotometer. The procedure was carried out at 37°C with the use of endotoxin-free materials.

### hGH activity assay

The bioactivity of recombinant hGH was tested using Nb2-11 cells (Sigma-Aldrich). The cells were maintained in Fischer's medium (Wellgene, Daegu, Korea) supplemented with 10% fetal bovine serum (Biowest, Nuaillé, France), 10% horse serum (Gibco/Invitrogen, Carlsbad, CA), and 1% penicillin-streptomycin (Gibco/Invitrogen) in a 37°C humidified incubator containing 5% CO_2_. Growing cells were harvested and transferred to starvation medium without fetal bovine serum for 24 h to slow down the rate of cell division. For the activity assay, approximately 4×10^4^ cells were seeded onto a 96 well-plate containing 100 µL starvation medium in the presence of different concentrations of purified or commercial hGH (Cell Guidance Systems, Babraham Research Park, Cambridge, UK). Each sample was performed in triplicate. Cell growth was evaluated using a water-soluble tetrazolium salt (WST-1) assay (Takara, Kyoto, Japan) after 18 h of incubation with the hormone.

### Statistics

In this study, the dose-response proliferation of Nb2-11 cells was standardized by a non-linear regression analysis. The data were fitted using the following equation and Microsoft Excel software:
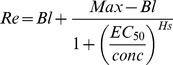
(1)where *Re* is the response of the cells, *Max* is the maximum response, *Bl* is the baseline at low concentration, *conc* is the concentration of the protein, and *Hs* is the Hill coefficient of stimulation. All data are presented as the mean ± standard error (SE) of n ≥3 of 2 independent experiments. GraphpadPrism 5 software (GraphPad, San Diego, CA) was used for statistical analyses.

## Results

### Construction of hGH plasmids and solubility testing

**Figure 1:**
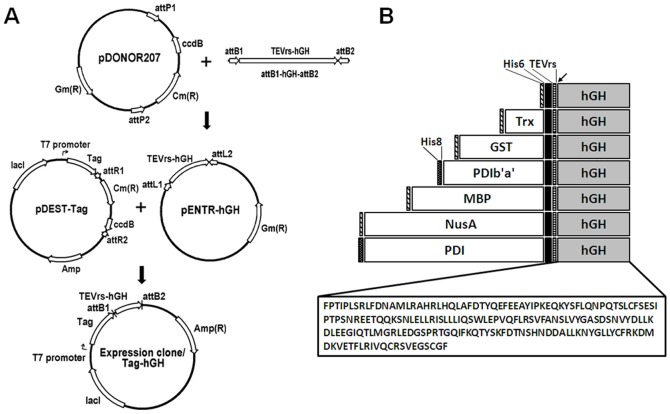
Schematic representation of domain structure and generation of the MBP-hGH construct. (A) Vector map of pHMGWA-hGH using the gateway cloning method. Expression of the fusion proteins in *E. coli* is controlled by the IPTG-inducible T7 promoter, with ampicillin as the selection marker. (B) Schematic structure of the seven fusion proteins His6-, Trx-, GST-, MBP-, NusA-, PDIb′a′-, and PDI-GH (total size). The arrows indicate the TEV protease cleavage site.

To test the effects of the various tags on the expression level and solubility of hGH in *E. coli*, we constructed seven plasmids that would express hGH with N-terminal His6, Trx, GST, MBP, NusA, PDI, or PDIb′a′ tags (Fig. 1). TEVrs was inserted between the tag and hGH to separate them during purification. To facilitate purification, His6 was attached at the N-terminus of Trx, GST, MBP, and NusA, and His8 was attached at the N-terminus of PDI and PDIb′a′ (Fig. 1B). The sequence of hGH and TEVrs was codon-optimized for *E. coli* expression. To facilitate subcloning, BP and LR recombinations were used. The sequences of the seven plasmids were confirmed and the seven plasmids were transformed into BL21(DE3) *E. coli* strain.

**Figure 2:**
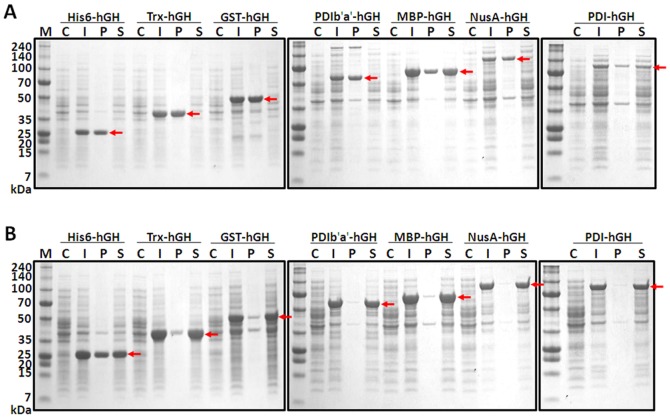
Expression analysis of tagged hGH in *E. coli*. Expression of full-length hGH was induced by 0.5 mM IPTG at 37°C (A) and 18°C (B). Arrows indicate hGH proteins fused with each tag. M, molecular weight marker; C, total cell protein before IPTG induction as a negative control; I, total cell protein after IPTG induction; P, pellet fraction after cell sonication; S, soluble supernatant after cell sonication.

Expression of hGH was driven by the T7 promoter, which is induced by the addition of IPTG. The expression level and solubility varied according to the tag and temperature. For example, at 37°C, the expression level of hGH with His6 was 64%, but the solubility was only 2.3%. However, MBP and PDI tags increased the solubility to up to 70% (Fig. 2A and Table 1). Addition of MBP and Trx tags increased the expression level to more than 80%. Lowering the expression temperature to 18°C increased the expression level of all tag-fused hGHs, especially for Trx-hGH, NusA-hGH and PDI-hGH to more than 70% (Fig. 2B and Table 1). More dramatically, the solubility of all fusion proteins was improved by reducing the expression temperature. Trx-, MBP-, NusA-, PDIb′a′-, and PDI-tagged hGH showed almost complete solubilization, whereas the solubility of His6-tagged hGH was 58%. Among the seven tags, Trx, MBP, and PDIb′a′ were chosen as fusion partners of hGH for further purification based on their expression level, solubility, and tag size to facilitate purification.

**Table 1:** Expression level and solubility of hGH with seven different tags.

	Tag	Tag size (kDa)	Fusion protein size (kDa)	Expression level (%)		Solubility (%)	
				37°C	18°C	37°C	18°C
**hGH (22 kDa)**	His6	0.8	25.6	63.9	68.3	2.3	58.0
	Trx	11.7	37.4	82.0	88.1	0.8	94.2
	GST	25.6	51.3	67.8	69.6	1.5	94.8
	MBP	40.2	65.9	81.2	87.8	67.9	98.8
	NusA	54.7	80.5	35.2	72.3	8.2	97.6
	PDIb′a′	30.6	56.6	54.0	75.0	2.7	98.4
	PDI	54.8	80.8	32.7	75.8	68.2	98.2

### Purification of recombinant hGH

**Figure 3:**
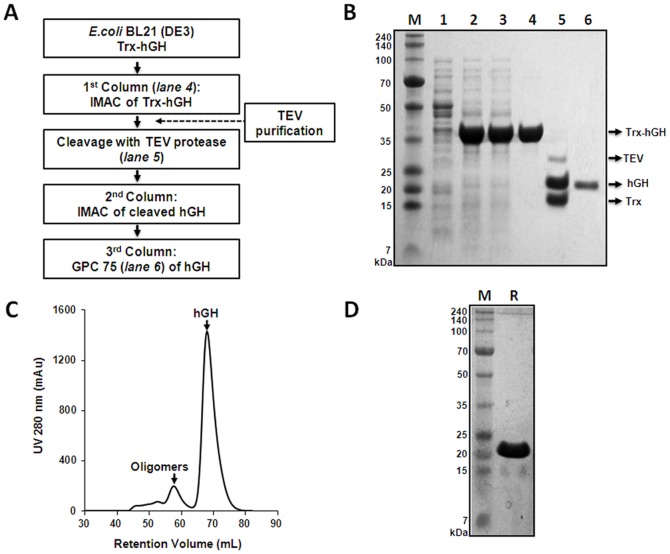
hGH purification from Trx-hGH expressed in *E. coli*. (A) Flowchart of the purification. (B) Trx-hGH was purified from *E. coli* with a combination of IMAC and gel filtration chromatography. M, molecular weight marker; lane 1, total cell protein before IPTG induction as a negative control; lane 2, total cell protein treated with IPTG; lane 3, soluble fraction after cell sonication; lane 4, Trx-hGH fusion protein purified using IMAC (37.4 kDa); lane 5, Trx tag cleavage with TEV protease: Trx (15.4 kDa) and hGH (22 kDa); lane 6, final purified hGH (22 kDa). Lane 5 shows that the Trx tag was almost completely cleaved. (C) Gel filtration chromatogram of PDIb′a′-hGH after second IMAC. hGH and oligomers were separated by their sizes. (D) Purity of final product hGH was evaluated by silver staining. M, molecular weight marker; hGH: final product in non-reducing conditions.

hGHs from MBP-hGH and PDIb′a′-hGH were purified by the same method using the same buffers (Fig. S1 in File S1 and Fig. S2 in File S1), while hGH from Trx-hGH was purified by the same method but with a slight variation in buffer constituents (Fig. 3). The cell lysate from Trx-hGH was precipitated under salt conditions during cell sonication, but resulted in a muddy milk color which indicates precipitation in 500 mM of NaCl or 5–10 mM of MgCl_2_. Therefore, unlike MBP-hGH or PDIb′a′-hGH, Trx-hGH was sonicated in a buffer without NaCl. The stability of Trx-hGH according to NaCl concentration was also tested for the supernatant after sonication and for the eluate after the first IMAC (Table 2 and Table 3). Precipitation of the Trx-hGH eluate after the first IMAC began at 10 mM of NaCl (Table 3). Thus, Trx-hGH was purified in the absence of NaCl before Trx tag cleavage. hGH was initially purified with IMAC. After the washing step, the bound fusion protein was eluted with 100 mM (Trx-hGH) or 1 M imidazole (MBP-hGH, PDIb′a′-hGH). During this step, the purity was found to be more than ∼95%. Then, the protein sample was dialyzed into TEV standard buffer for tag cleavage because high salt concentration can interfere with TEV activity [35]. Finally, the TEV protease was added to remove the tag. Cleavage conditions of 1∶30 and 1∶10 (w/w) ratio of TEV:protein were tested for PDIb′a′-hGH at 18°C for 2 hours, and even at a ratio of 1∶30 (w/w), PDIb′a′ had almost completely separated. Therefore, the same cleavage condition was used for MBP-hGH and PDIb′a′-hGH. For Trx-hGH, a ratio of 1∶10 (w/w) of TEV:sample at 4°C for 4 hours was enough for almost complete cleavage. SDS-PAGE revealed that the fusion protein was cleaved almost completely in only 2 hours of incubation at 18°C (MBP-hGH, PDIb′a′-hGH; Fig. S1 in File S1, lane 5, Fig. S2 in File S1, lane 5) and cleaved completely at 4°C for 4 hours (Trx-hGH; Fig. 3, lane 5). Cleaved hGH was separated from the tag and TEV by IMAC. Interestingly, hGH from Trx-hGH did not bind to, but rather passed through the column, but hGHs from MBP-hGH and PDIb′a′-hGH bound nonspecifically to the column in 500 mM NaCl and were eluted fully with 50 mM of imidazole. Finally, Superdex-75 molecular sieve chromatography was applied to separate the purified hGH monomer from the dimer or oligomer. Two peaks, an earlier small peak and later large peak, were found in the chromatogram (Fig. 3C). The large peak was identified as the hGH monomer and the small earlier peak contained the dimer with small oligomers based on the native gel run (data not shown). Therefore, gel filtration nicely separated the monomer from oligomers of hGH. However, with no DTT in the buffer, a small amount of dimer was eluted along with monomer hGH; therefore, to obtain pure monomer hGH, 2.5 mM of DTT was added to the buffer during gel filtration and then removed from the final protein sample by dialysis.

**Table 2:** Stability test for Trx-hGH supernatant.

NaCl concentration (mM)	0	10	50	100	500	1000
Protein concentration (mg/mL)	0.57	0.58	0.59	0.60	0.47	0.45
Precipitation (%)	5	3	1.7	0	21.7	25

**Table 3:** Stability test for the 1^st^IMAC eluate of Trx-hGH.

NaCl concentration (mM)	0	10	50	100	500	1000
Protein concentration (mg/mL)	0.66	0.61	0.48	0.41	0.30	0.22
Precipitation (%)	0	7.6	27.3	37.9	54.5	66.7

Silver staining under reducing conditions indicated that the final product contained a single band corresponding to 22 kDa hGH (Fig. 3D) with a purity after the final step of 99%. In Tables 4, S1 in File S1, and S2 in File S1, the purification of hGH from Trx-hGH, MBP-hGH, and PDIb′a′-hGH expressed in *E. coli*, respectively, is shown. Approximately 37 mg, 11.7 mg, and 6.7 mg of pure hGHs were obtained from 2.01 g, 1.8 g, and 1.6 g wet weight cells of Trx-hGH, MBP-hGH, and PDIb′a′-hGH, respectively, from 500 mL of cell culture in general. The final amount of purified hGH from PDIb′a′-hGH was considerably lower than that from Trx-hGH or MBP-hGH. However, the yield of hGH from PDIb′a′-hGH was about 10% higher than that of hGH from Trx-hGH and MBP-hGH. The endotoxin levels of the final purified hGHs from Trx-hGH, MBP-hGH, and PDIb′a′-hGH were 0.051 EU/µg, 0.058 EU/µg, and 0.052 EU/µg, respectively, and these values are much lower than 1 EU/µg, the criterion required for a safe protein product.

**Table 4:** Purification table of hGH from Trx-hGH expressed in *E. coli* at 18°C.

Purification step	Volume (mL)	Concentration (mg/mL)	Total protein (mg)	Purity (%)	hGH (mg)	Yield (%)
Bacterial culture	500	-	2010 (pellet)	-		-
Supernatant	100	4.55	455	88	235.5	100
1^st^ IMAC eluate	50	3.5	175	98	100.9	43
2^nd^ IMAC eluate	150	0.52	78	97	75.7	32
GPC	150	0.25	37.5	99	37.1	16

### MALDI-TOF MS analysis

**Figure 4:**
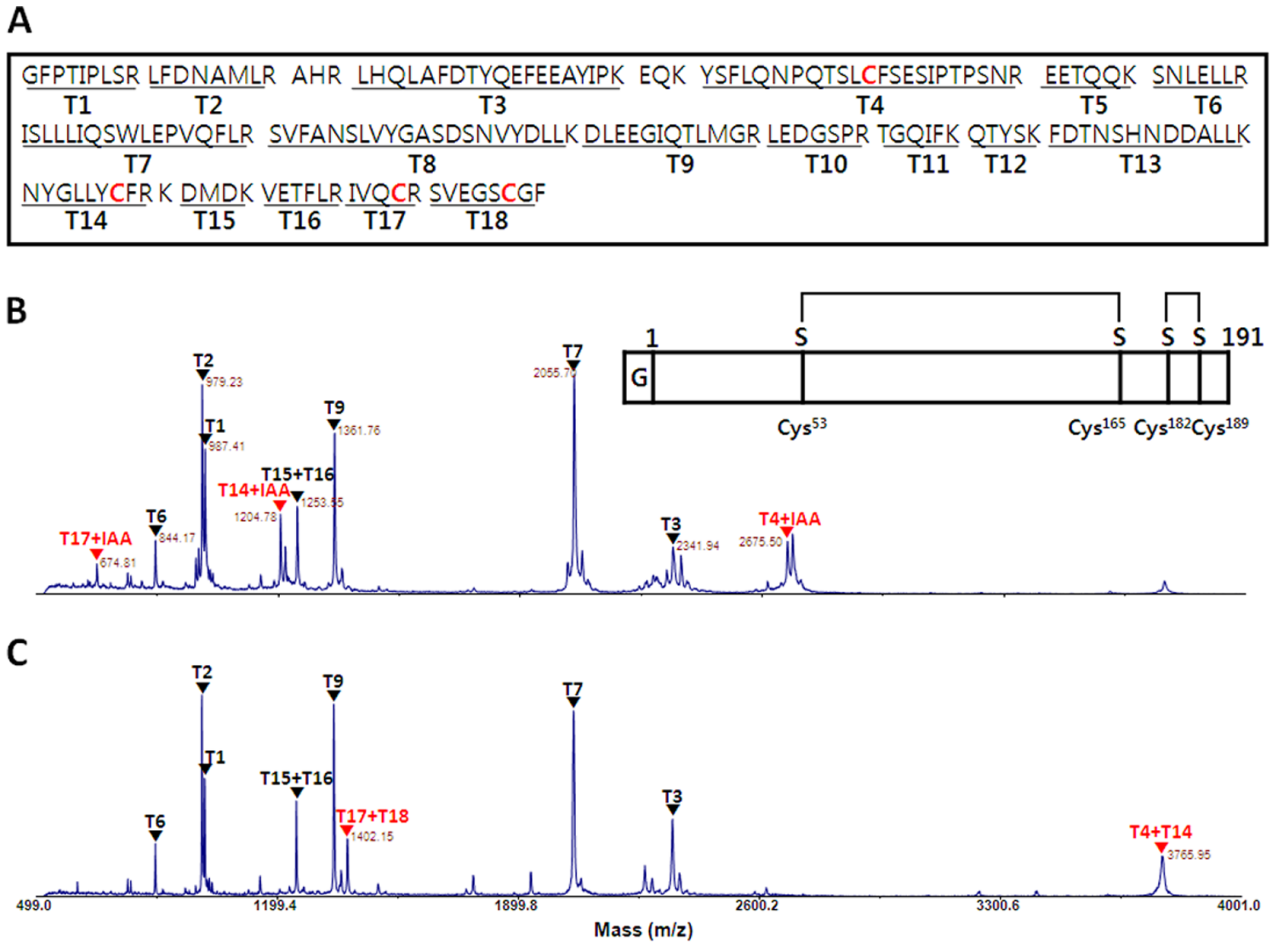
MALDI-TOF MS analysis of purified hGH from Trx-hGH in *E. coli* (no DTT during purification). (A) Tryptic peptide map of hGH (191 aa). (B) MALDI-TOF MS for purified hGH in reducing conditions. (C) MALDI-TOF MS for the purified hGH in non-reducing conditions. MALDI-TOF MS analysis of reduced and non-reduced hGH obtained from MBP-hGH and PDIb′a′-hGH showed similar results (data not shown).

To confirm the identity of the purified protein and presence of correct disulfide bonds, MALDI-TOF MS analysis was performed in reduced and non-reduced hGH cleaved from 5 µg of hGH (Fig. 4). The results showed that most of the peaks corresponded to the masses of trypsin-digested fragments of hGH (Fig. 4B). Also, the data confirmed that four cysteine residues of hGH correctly participated in intra-molecular disulfide bond formation under non-reducing conditions (Fig. 4C). The alkylating agent, IAA, was used to block disulfide bond formation in reducing conditions using DTT. IAA commonly binds covalently to the thiol group of cysteine. We identified 3 peptide fragments that each has one Cys alkylated by IAA, T4 (Cys53), T14 (Cys165), and T17 (Cys182) (Fig. 4B). Somehow, T18 (Cys189) was not detected. However, in non-reducing conditions, all peaks alkylated with IAA disappeared and two new peaks corresponding to the exact size of disulfide-bonded cysteines between T17 and T18 (1,402 Da) and between T4 and T14 (3,766 Da) appeared (Fig. 4C). This demonstrated that two correct intra-molecular disulfide bonds are present in hGH.

### Biological activity

**Figure 5:**
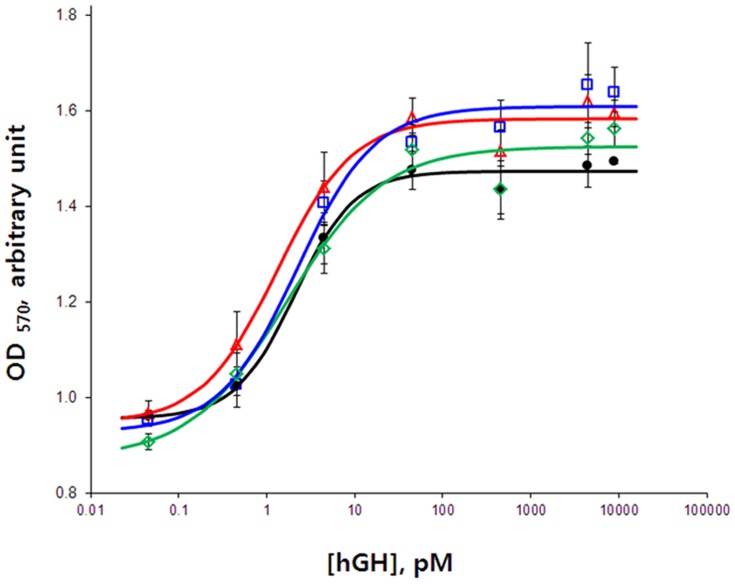
Cell proliferation assay of purified hGH in the Nb2-11 cell line. Dose-response proliferation curve of Nb2-11 cells by exposure to different concentrations of purified hGH and commercial hGH.•, commercial hGH; △, hGH from Trx-hGH; ◊, hGH from Trx-hGH in presence of DTT; □, hGH from MBP-hGH.

The growth of Nb2-11 cells is stimulated by mammalian lactogens. Because hGH is a lactogenic hormone, the cell growth is an effective tool to determine the activity of recombinant hGH [36], [37]. Various concentrations of purified and commercial hGH were prepared to evaluate the growth-promoting effect of the hormone in cells (Fig. 5). WST-1 assay results showed that commercial and purified hGHs stimulated Nb2-11 cell growth in a dose-dependent manner, with no significant differences between purified hGH and commercial hGH-treated cells. The dose-response curves showed a sigmoidal pattern. We compared the activities of purified hGHs from Trx-hGH and MBP-hGH to a commercial hGH. We also compared hGHs purified from Trx-hGH in the presence or absence of DTT during purification. All dose-response curves increased exponentially in the range from 0.45 pM to 45 pM. The EC_50_s of commercial hGH, hGH from Trx-hGH with or without DTT during purification, and hGH from MBP-hGH were 2.07±0.52 pM, 1.67±0.69 pM, 1.30±0.50 pM, and 2.19±0.1 pM, respectively, with Hill coefficients of 1.28±0.09, 0.79±0.24, 1.01±0.07, and 0.99±0.2, respectively. The EC_50_ of purified hGH from MBP fusion protein exhibited a similar response to that of commercial hGH, whereas the biological activity of hGH from Trx fusion protein was slightly higher. However, hGH from MBP-hGH resulted in a continuously increased pattern of cellular response, whereas the activities of the others decreased from 45 pM to 0.45 nM and then increased again.

## Discussion

The aim of this study was to assess the effect of various tags on the solubility of hGH overexpressed in the cytoplasm of *E. coli* as a method to increase the production efficiency of hGH. To do this, we attached seven tags—His6, Trx, GST, MBP, NusA, PDI, and PDIb′a′—at the N-terminus of hGH and measured the expression level and solubility of the resulting fusion proteins. While all the constructs showed good expression levels, the solubilities differed significantly depending on the tag and expression temperature (Table 1). At 37°C, only MBP and PDI showed solubility higher than 50%. However, at lower temperature, all the fusion proteins except the His6 tag showed more than 90% solubility. These results indicate that a lower expression temperature increases the solubility of hGH in *E. coli* and is consistent with a recent report using His tag [25]. Based on the protein expression level, solubility, and size of the fusion protein, we selected three tags, Trx, MBP, and PDIb′a′, for further purification. Trx is known to serve as a general protein disulfide oxidoreductase by facilitating the reduction of other proteins by cysteine thiol-disulfide exchange [38], [39]. MBP acts as a general molecular chaperone to promote the proper folding of its fusion partner [40]. In addition, MBP is thought to bind to hydrophobic residues present on non-folded proteins, thereby preventing aggregation [41]. MBP is also resistant to proteolysis and may protect its partner protein from degradation [42]. PDI is an enzyme that catalyzes disulfide formation and isomerization, and acts as a chaperone to inhibit aggregation [43]. Although expression and solubilization by PDI were similar to those of PDIb′a′, PDIb′a′ was chosen because it is a smaller functional domain of PDI, as confirmed in our previous study (data not shown) and thus easier to use. Due to the function of PDI as a disulfide isomerase and chaperone, we expected the purification of hGH with PDIb′a′ to best perform among the three tags. However, the amount of final purified protein from PDIb′a′-hGH was the lowest among the three different hGH fusion proteins from the same volume of culture media. However, the purification yield (28%) of hGH from PDIb′a′-hGH was the highest among the three tagged hGHs (Tables 4, S1 in File S1, and S2 in File S1). Therefore, while the amount was lower, PDIb′a′ had the highest efficiency in hGH production.

Generally, many buffers used in protein purification contain NaCl to maintain the solubility of proteins and to mimic physiological conditions [44]. However, interestingly, hGH fused with Trx tag protein precipitated in the buffer containing salts, such as NaCl or MgCl_2_, at sonication. Even a clear supernatant and the first IMAC eluate precipitated when NaCl was added. In agreement with these results, Kim *et al.* reported that the solubility of hGH somewhat decreased by the addition of NaCl or KCl [25]. The authors explained that the addition of salt leads to increased hydrophobicity on the protein surface, resulting in protein aggregation through hydrophobic interactions, a process referred to as ‘salting out’ [25], [45]. In our experiments, MBP- and PDIb′a′-tagged hGHs were soluble in a buffer containing NaCl, and this may be because MBP (40.2 kDa) and PDIb′a′ (30.6 kDa) are big enough to prevent increases in surface hydrophobicity of hGH (22 kDa) with the addition of salt compared to Trx (11.7 kDa) or His tag, which are smaller than hGH [25].

The 22-kDa hGH isoform is a non-glycosylated single chain polypeptide that consists of 191 amino acids with two intra-molecular disulfide bonds between Cys^53^-Cys^165^ and Cys^182^-Cys^189^ [7]. Crystal structure analysis reveals that hGH is an anti-parallel four-helix bundle molecule with an orientation of “up(α1)-up(α2)-down(α3)-down(α4)” in the helical segments [7], [46], [47]. Disulfide bonds play a very important role in protein folding, stability, and protein bioactivity, but early studies revealed that disulfide bonds were not important for hGH bioactivity [48], [49]. Nevertheless, evolutionary conserved data [50], [51] have shown that four cysteins–Cys^53^, Cys^165^, Cys^182^, and Cys^189^–are well-conserved sequences and confirmed the importance of both disulfide bridges for GH protein stability. In addition, with advances in biotechnology, recent studies suggest that the disulfide bond Cys^53^-Cys^165^ is essential for the full activity of hGH through the Jak2/Stat5 signaling pathway, whereas the disulfide bond Cys^182^-Cys^189^ is required for protein stability and optimal binding to the hGH receptor [52], [53]. In our MS analysis (Fig. 4), two intra-molecular disulfide-bonded fragments were detected. This indicates that the two functional intra-molecular disulfide bonds are present in hGH, meaning that hGH purified from *E. coli* possesses full biological activity comparable to the native form in the human body. However, we could not clearly detect the two intra-molecular disulfide bonded peaks in hGH purified in the presence of DTT. While it was removed from the final protein sample by dialysis, residual DTT may have affected disulfide bridges by exposing the two intra-molecular disulfide bonds, Cys^53^-Cys^165^, which occur between the flexible region (between α1 and α2) and alpha helix 4, and Cys^182^-Cys^189^ placed in the end of α4 with a flexible C-terminus. The GH oligomers have been reported to have from moderately reduced to full bioactivity [54]. Interestingly, the biological activities of our hGHs purified with or without DTT were almost the same, suggesting that low amounts of dimer hGH do not affect bioactivity. However, the effect of the disulfide bonds on biological activity should be confirmed using higher sensitivity mass technology.

*E. coli* does not have its own protein glycosylation machinery, meaning that hGH expressed in *E. coli*, as in human cells [24], is non-glycosylated. Therefore, the non-glycosylation of hGH from *E. coli* does not pose a problem for its functional activity. However, glycosylation generally increases the half-life of a protein circulating in the blood by protecting the protein from proteases. If hGH is used *in vivo* and requires an extended half-life than the native form, glycosylation-like modifications such as PEGylation or chitosanylation can be attempted.

In conclusion, we here described the overexpression of hGH in a soluble form using Trx, MBP, and PDIb′a′ fusion proteins by lowering the expression temperature in the cytoplasm of *E. coli* cells. We also demonstrated simple purification of hGH using affinity and gel filtration chromatography. We obtained more than 37 mg of pure hGH from 2 g of 500 mL cell culture. In addition, we successfully purified TEV protease, which was used to cut the fusion protein and separate pure hGH. Finally, we confirmed the presence of two correct intra-molecular disulfide bonds in the purified hGH using MALDI-TOF MS and demonstrated biological activity of purified hGH using Nb2-11 cells. Purified hGH showed slightly better biological activity compared to commercial hGH. These results show that mass production of bio-drugs can be achieved by an efficient and cost-effective technique.

## Supporting Information

File S1 **Supporting tables and figures.** **Table S1,** Purification table of hGH from MBP-hGH expressed in *E. coli* at 18°C. **Table S2,** Purification table of hGH from PDIb′a′-hGH expressed in *E. coli* at 18°C. **Figure S1,** hGH purification from MBP-hGH expressed in *E. coli*. **Figure S2,** hGH purification from PDIb′a′-hGH expressed in *E. coli*.
(DOCX)

## References

[1] Xu J, Messina JL (2009) Crosstalk between growth hormone and insulin signaling. VitamHorm 80: 125–153. 10.1016/S0083-6729(08)00606-7 19251037

[2] Le Roith D, Bondy C, Yakar S, Liu JL, Butler A (2001) The somatomedin hypothesis: 2001. Endocr Rev 22: 53–74. 11159816 10.1210/edrv.22.1.0419

[3] Yarasheski KE, Campbell JA, Smith K, Rennie MJ, Holloszy JO, et al (1992) Effect of growth hormone and resistance exercise on muscle growth in young men. Am J Physiol 262: E261–267. 1550219 10.1152/ajpendo.1992.262.3.E261

[4] Nilsson A, Ohlsson C, Isaksson OG, Lindahl A, Isgaard J (1994) Hormonal regulation of longitudinal bone growth. Eur J ClinNutr 48 Suppl 1: S150–158 discussion S158–160. 10.1007/BF02558817 8005082

[5] Gu Y, Zou Y, Aikawa R, Hayashi D, Kudoh S, et al (2001) Growth hormone signalling and apoptosis in neonatal rat cardiomyocytes. Mol Cell Biochem 223: 35–46. 11681720 10.1023/a:1017941625858

[6] Jeay S, Sonenshein GE, Postel-Vinay MC, Kelly PA, Baixeras E (2002) Growth hormone can act as a cytokine controlling survival and proliferation of immune cells: new insights into signaling pathways. Mol Cell Endocrinol 188: 1–7. 11911939 10.1016/s0303-7207(02)00014-x

[7] Kopchick JJ (2003) History and future of growth hormone research. Horm Res 60 Suppl 3: 103–112. 10.1159/000074510 14671406

[8] Ayyar VS (2011) History of growth hormone therapy. Indian J EndocrinolMetab 15 Suppl 3: S162–165. 10.4103/2230-8210.84852 PMC3183530 22029019

[9] Tritos NA, Mantzoros CS (1998) Recombinant human growth hormone: old and novel uses. Am J Med 105: 44–57. 9688021 10.1016/s0002-9343(98)00135-1

[10] Bolar K, Hoffman AR, Maneatis T, Lippe B (2008) Long-term safety of recombinant human growth hormone in turner syndrome. J ClinEndocrinolMetab 93: 344–351. 10.1210/jc.2007-1723 18000090

[11] Franklin SL, Geffner ME (2009) Growth hormone: the expansion of available products and indications. EndocrinolMetabClin North Am 38: 587–611. 10.1016/j.ecl.2009.06.006 19717006

[12] Martial JA, Hallewell RA, Baxter JD, Goodman HM (1979) Human growth hormone: complementary DNA cloning and expression in bacteria. Science 205: 602–607. 377496 10.1126/science.377496

[13] Catzel D, Lalevski H, Marquis CP, Gray PP, Van Dyk D, et al (2003) Purification of recombinant human growth hormone from CHO cell culture supernatant by Gradiflow preparative electrophoresis technology. Protein ExprPurif 32: 126–134. 10.1016/j.pep.2003.07.002 14680949

[14] Calik P, Orman MA, Celik E, Halloran SM, Calik G, et al (2008) Expression system for synthesis and purification of recombinant human growth hormone in Pichiapastoris and structural analysis by MALDI-ToF Mass Spectrometry. BiotechnolProg 24: 221–226. 10.1021/bp070294t 18186643

[15] Apte-Deshpande A, Rewanwar S, Kotwal P, Raiker VA, Padmanabhan S (2009) Efficient expression and secretion of recombinant human growth hormone in the methylotrophic yeast Pichiapastoris: potential applications for other proteins. BiotechnolApplBiochem 54: 197–205. 10.1042/BA20090179 19814714

[16] Lan H, Nie Z, Liu Y, Lv Z, Quan Y, et al (2010) In vivo bioassay of recombinant human growth hormone synthesized in B. mori pupae. J Biomed Biotechnol 2010: 306462. 20339512 10.1155/2010/306462 PMC2842897

[17] Shin NK, Kim DY, Shin CS, Hong MS, Lee J, et al (1998) High-level production of human growth hormone in Escherichia coli by a simple recombinant process. Journal of Biotechnology 62: 143–151. 9706704 10.1016/s0168-1656(98)00054-6

[18] Patra AK, Mukhopadhyay R, Mukhija R, Krishnan A, Garg LC, et al (2000) Optimization of inclusion body solubilization and renaturation of recombinant human growth hormone from Escherichia coli. Protein ExprPurif 18: 182–192. 10.1006/prep.1999.1179 10686149

[19] Solomon G, Reicher S, Gussakovsky EE, Jomain JB, Gertler A (2006) Large-scale preparation and in vitro characterization of biologically active human placental (20 and 22K) and pituitary (20K) growth hormones: placental growth hormones have no lactogenic activity in humans. Growth Hormone and IGF Research 16: 297–307. 17010651 10.1016/j.ghir.2006.07.002

[20] Sonoda H, Sugimura A (2008) Improved solubilization of recombinant human growth hormone inclusion body produced in Escherichia coli. BiosciBiotechnolBiochem 72: 2675–2680. 10.1271/bbb.80332 18838803

[21] Singh SM, Sharma A, Panda AK (2009) High throughput purification of recombinant human growth hormone using radial flow chromatography. Protein ExprPurif 68: 54–59. 10.1016/j.pep.2009.05.014 19500673

[22] Fahnert B, Lilie H, Neubauer P (2004) Inclusion bodies: formation and utilisation. Advances in Biochemical Engineering/Biotechnology 89: 93–142. 15217157 10.1007/b93995

[23] Becker GW, Hsiung HM (1986) Expression, secretion and folding of human growth hormone in Escherichia coli. Purification and characterization. FEBS Letters 204: 145–150. 3527743 10.1016/0014-5793(86)81403-x

[24] Sockolosky JT, Szoka FC (2013) Periplasmic production via the pET expression system of soluble, bioactive human growth hormone. Protein ExprPurif 87: 129–135. 10.1016/j.pep.2012.11.002 PMC3537859 23168094

[25] Kim MJ, Park HS, Seo KH, Yang HJ, Kim SK, et al (2013) Complete solubilization and purification of recombinant human growth hormone produced in Escherichia coli. PLoS One 8: e56168. 23409149 10.1371/journal.pone.0056168 PMC3567055

[26] Koo TY, Park TH (2007) Expression of recombinant human growth hormone in a soluble form in Escherichia coli by slowing down the protein synthesis rate. J MicrobiolBiotechnol 17: 579–585. 18051267

[27] Wang S, Shen M, Xu Y, Chen F, Chen M, et al (2013) Rational and efficient preparation of a chimeric protein containing a tandem dimer of thrombopoietin mimetic peptide fused to human growth hormone in Escherichia coli. Applied Microbiology and Biotechnology 97: 2885–2894. 23149755 10.1007/s00253-012-4553-7

[28] Hartley JL, Temple GF, Brasch MA (2000) DNA cloning using in vitro site-specific recombination. Genome Res 10: 1788–1795. 11076863 10.1101/gr.143000 PMC310948

[29] Busso D, Delagoutte-Busso B, Moras D (2005) Construction of a set Gateway-based destination vectors for high-throughput cloning and expression screening in Escherichia coli. Analytical Biochemistry 343: 313–321. 15993367 10.1016/j.ab.2005.05.015

[30] Song JA, Koo BK, Chong SH, Kwak J, Ryu HB, et al (2013) Expression and purification of biologically active human FGF2 containing the b′a′ domains of human PDI in *Escherichia coli* . Applied Biochemistry and Biotechnology 170: 67–80. 23471584 10.1007/s12010-013-0140-3

[31] Bradford MM (1976) A rapid and sensitive method for the quantitation of microgram quantities of protein utilizing the principle of protein-dye binding. Anal Biochem 72: 248–254. 942051 10.1016/0003-2697(76)90527-3

[32] Kapust RB, Tozser J, Fox JD, Anderson DE, Cherry S, et al (2001) Tobacco etch virus protease: mechanism of autolysis and rational design of stable mutants with wild-type catalytic proficiency. Protein Engineering 14: 993–1000. 11809930 10.1093/protein/14.12.993

[33] Lee JW, Helmann JD (2006) The PerR transcription factor senses H2O2 by metal-catalysedhistidine oxidation. Nature 440: 363–367. 16541078 10.1038/nature04537

[34] Jung AS, Koo BK, Chong SH, Kim K, Choi DK, et al (2013) Soluble Expression of Human Leukemia Inhibitory Factor with Protein Disulfide Isomerase in Escherichia coli and Its Simple Purification. PLoS One 8: e83781. 24358310 10.1371/journal.pone.0083781 PMC3865251

[35] Waugh DS (2011) An overview of enzymatic reagents for the removal of affinity tags. Protein ExprPurif 80: 283–293. 10.1016/j.pep.2011.08.005 PMC3195948 21871965

[36] Tanaka T, Shiu RP, Gout PW, Beer CT, Noble RL, et al (1980) A new sensitive and specific bioassay for lactogenic hormones: measurement of prolactin and growth hormone in human serum. J ClinEndocrinolMetab 51: 1058–1063. 10.1210/jcem-51-5-1058 7419681

[37] Walsh RJ, Mangurian LP, Posner BI (1990) The distribution of lactogen receptors in the mammalian hypothalamus: an in vitro autoradiographic analysis of the rabbit and rat. Brain Res 530: 1–11. 2176913 10.1016/0006-8993(90)90651-q

[38] Holmgren A (1985) Thioredoxin. Annu Rev Biochem 54: 237–271. 3896121 10.1146/annurev.bi.54.070185.001321

[39] Nordberg J, Arner ES (2001) Reactive oxygen species, antioxidants, and the mammalian thioredoxin system. Free RadicBiol Med 31: 1287–1312. 10.1016/s0891-5849(01)00724-9 11728801

[40] Bach H, Mazor Y, Shaky S, Shoham-Lev A, Berdichevsky Y, et al (2001) Escherichia coli maltose-binding protein as a molecular chaperone for recombinant intracellular cytoplasmic single-chain antibodies. Journal of Molecular Biology 312: 79–93. 11545587 10.1006/jmbi.2001.4914

[41] Kapust RB, Waugh DS (1999) Escherichia coli maltose-binding protein is uncommonly effective at promoting the solubility of polypeptides to which it is fused. Protein Science 8: 1668–1674. 10452611 10.1110/ps.8.8.1668 PMC2144417

[42] Park C, Zhou S, Gilmore J, Marqusee S (2007) Energetics-based protein profiling on a proteomic scale: identification of proteins resistant to proteolysis. J MolBiol 368: 1426–1437. 10.1016/j.jmb.2007.02.091 PMC2857998 17400245

[43] Wilkinson B, Gilbert HF (2004) Protein disulfide isomerase. BiochimBiophysActa 1699: 35–44. 10.1016/j.bbapap.2004.02.017 15158710

[44] Structural Genomics Consortium China Structural Genomics Consortium Northeast Structural Genomics Consortium (2008) Graslund S, Nordlund P, et al (2008) Protein production and purification. Nat Methods 5: 135–146. 18235434 10.1038/nmeth.f.202 PMC3178102

[45] Baldwin RL (1996) How Hofmeister ion interactions affect protein stability. Biophys J 71: 2056–2063. 8889180 10.1016/S0006-3495(96)79404-3 PMC1233672

[46] Kasimova MR, Kristensen SM, Howe PW, Christensen T, Matthiesen F, et al (2002) NMR studies of the backbone flexibility and structure of human growth hormone: a comparison of high and low pH conformations. J MolBiol 318: 679–695. 10.1016/S0022-2836(02)00137-7 12054815

[47] De Palo EF, De Filippis V, Gatti R, Spinella P (2006) Growth hormone isoforms and segments/fragments: molecular structure and laboratory measurement. ClinChimActa 364: 67–76. 10.1016/j.cca.2005.06.009 16194529

[48] Dixon JS, Li CH (1966) Retention of the biological potency of human pituitary growth hormone after reduction and carbamidomethylation. Science 154: 785–786. 5919448 10.1126/science.154.3750.785

[49] Connors MH, Kaplan SL, Li CH, Grumbach MM (1973) Retention of biologic activity of human growth hormone in man after reduction and alkylation. J ClinEndocrinolMetab 37: 499–504. 10.1210/jcem-37-4-499 4742534

[50] Watahiki M, Yamamoto M, Yamakawa M, Tanaka M, Nakashima K (1989) Conserved and unique amino acid residues in the domains of the growth hormones. Flounder growth hormone deduced from the cDNA sequence has the minimal size in the growth hormone prolactin gene family. J BiolChem 264: 312–316. 2909523

[51] Cunningham BC, Wells JA (1989) High-resolution epitope mapping of hGH-receptor interactions by alanine-scanning mutagenesis. Science 244: 1081–1085. 2471267 10.1126/science.2471267

[52] Besson A, Salemi S, Deladoey J, Vuissoz JM, Eble A, et al (2005) Short stature caused by a biologically inactive mutant growth hormone (GH-C53S). J ClinEndocrinolMetab 90: 2493–2499. 10.1210/jc.2004-1838 15713716

[53] Junnila RK, Kopchick JJ (2013) Significance of the disulphide bonds of human growth hormone. Endokrynol Pol 64: 300–305. 24002958 10.5603/ep.2013.0009

[54] Baumann GP (2009) Growth hormone isoforms. Growth Horm IGF Res 19: 333–340. 19467614 10.1016/j.ghir.2009.04.011

