# Supplementary material for: Prokaryotic Soluble Overexpression and Purification of Bioactive Human Growth Hormone by Fusion to Thioredoxin, Maltose Binding Protein, and Protein Disulfide Isomerase
Source: PLoS One. 2014 Mar 10;9(3):e89038. doi: 10.1371/journal.pone.0089038 (PMC3948679; doi:10.1371/journal.pone.0089038)
Supplement: File S1 — Supporting tables and figures. Table S1, Purification table of hGH from MBP-hGH expressed in E. coli at 18°C. Table S2, Purification table of hGH from PDIb′a′-hGH expressed in E. coli at 18°C. Figure S1, hGH purification from MBP-hGH expressed in E. coli. Figure S2, hGH purification from PDIb′a′-hGH expressed in E. coli. (DOCX) [file pone.0089038.s001.docx]

**Supplementary Tables**

**Table S1. Purification table of hGH from MBP-hGH expressed in *E. coli* at 18**°**C**

| Purification step | Volume  (mL) | Concentration  (mg/mL) | Total protein  (mg) | Purity  (%) | hGH (mg) | Yield  (%) |
| --- | --- | --- | --- | --- | --- | --- |
| Bacterial culture | 500 | - | 1,800 (pellet) | - |  | - |
| Supernatant | 50 | 4.53 | 226.5 | 88 | 66.5 | 100 |
| 1^st^ IMAC eluate | 15 | 4.80 | 72.0 | 98 | 23.6 | 35 |
| 2^nd^ IMAC eluate | 20 | 0.92 | 18.3 | 96 | 17.6 | 26 |
| GPC | 56 | 0.21 | 11.8 | 99 | 11.7 | 18 |

**Table S2. Purification table of hGH from PDIb'a'-hGH expressed in *E. coli* at 18**°**C**

| Purification step | Volume  (mL) | Concentration  (mg/mL) | Total protein  (mg) | Purity  (%) | hGH (mg) | Yield  (%) |
| --- | --- | --- | --- | --- | --- | --- |
| Bacterial culture | 500 | - | 1,600 (pellet) | - |  | - |
| Supernatant | 100 | 0.82 | 82.0 | 75 | 24.0 | 100 |
| 1^st^ IMAC eluate | 40 | 1.01 | 40.4 | 98 | 15.4 | 64 |
| 2^nd^ IMAC eluate | 40 | 0.28 | 11.2 | 97 | 10.9 | 45 |
| GPC | 17 | 0.40 | 6.8 | 99 | 6.7 | 28 |

**Supplementary Figure Legends**

**Figure S1. hGH purification from MBP-hGH expressed in *E. coli*.** (A) Flowchart of the purification. (B) MBP-hGH was purified from *E. coli* with a combination of IMAC and gel filtration chromatography. M, molecular weight marker; lane 1, total cell protein before IPTG induction as negative control; lane 2, total cell protein treated with IPTG; lane 3, soluble fraction after cell sonication; lane 4, MBP-hGH fusion protein purified using IMAC (65.9 kDa); lane 5, MBP tag cleavage with TEV protease: MBP (43.9 kDa) and hGH (22 kDa); lane 6, final purified hGH. Lane 5 shows that the MBP tag was almost completely cleaved. (C) Gel filtration chromatogram of MBP-hGH after second IMAC. hGH and oligomers were separated by their sizes. (D) Purity of final product hGH was evaluated by silver staining. M, molecular weight marker; hGH: final product in non-reducing conditions.

**Figure S2. hGH purification from PDIb'a'-hGH expressed in *E. coli*.** (A) Flowchart of the purification. (B) PDIb'a'-hGH was purified from *E. coli* with a combination of IMAC and gel filtration chromatography. M, molecular weight marker; lane 1, total cell protein before IPTG induction as negative control; lane 2, total cell protein treated with IPTG; lane 3, soluble fraction after cell sonication; lane 4, PDIb'a'-hGH fusion protein purified using IMAC (56.6 kDa); lane 5, PDIb'a' tag cleavage with TEV protease: PDIb'a' (34.6 kDa) and hGH (22 kDa); lane 6, final purified hGH. Lane 5 shows that the PDIb'a' tag was almost completely cleaved. (C) Gel filtration chromatogram of PDIb'a'-hGH after second IMAC. hGH and oligomers were separated by their sizes. (D) Purity of final product hGH was evaluated by silver staining. M, molecular weight marker; hGH: final product in non-reducing conditions.


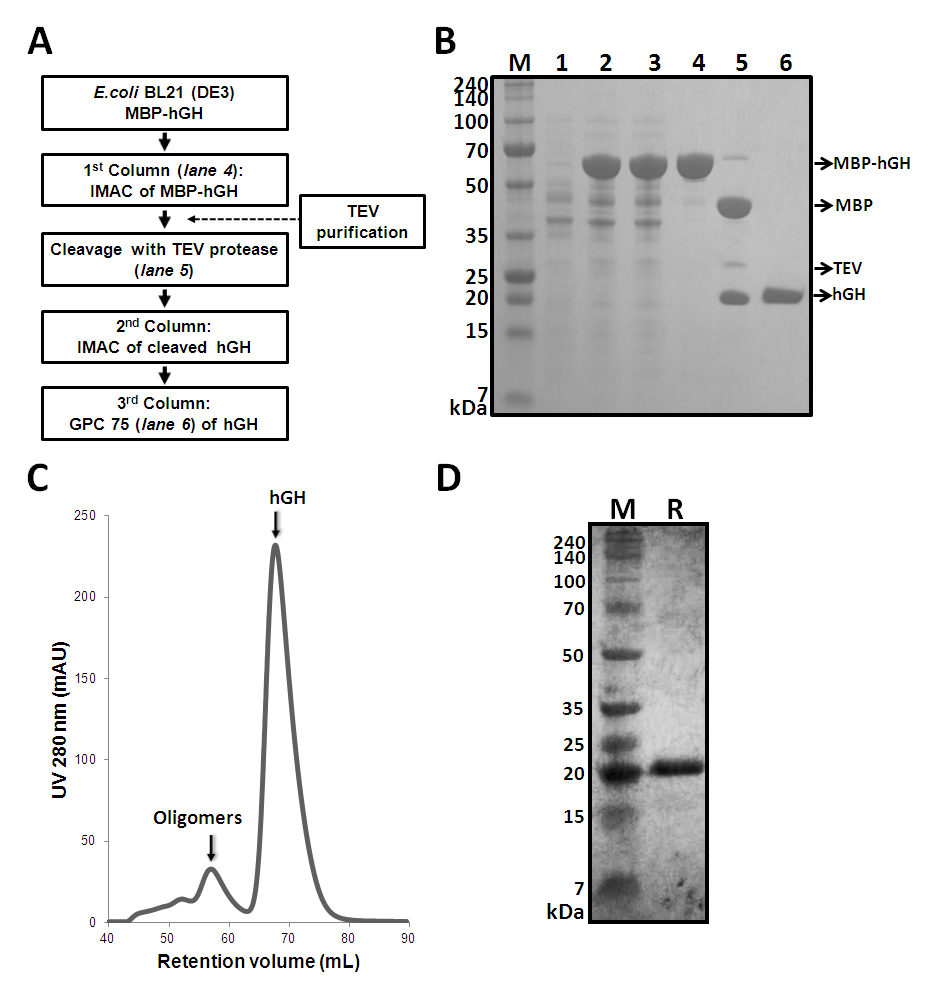


**Figure S1.**


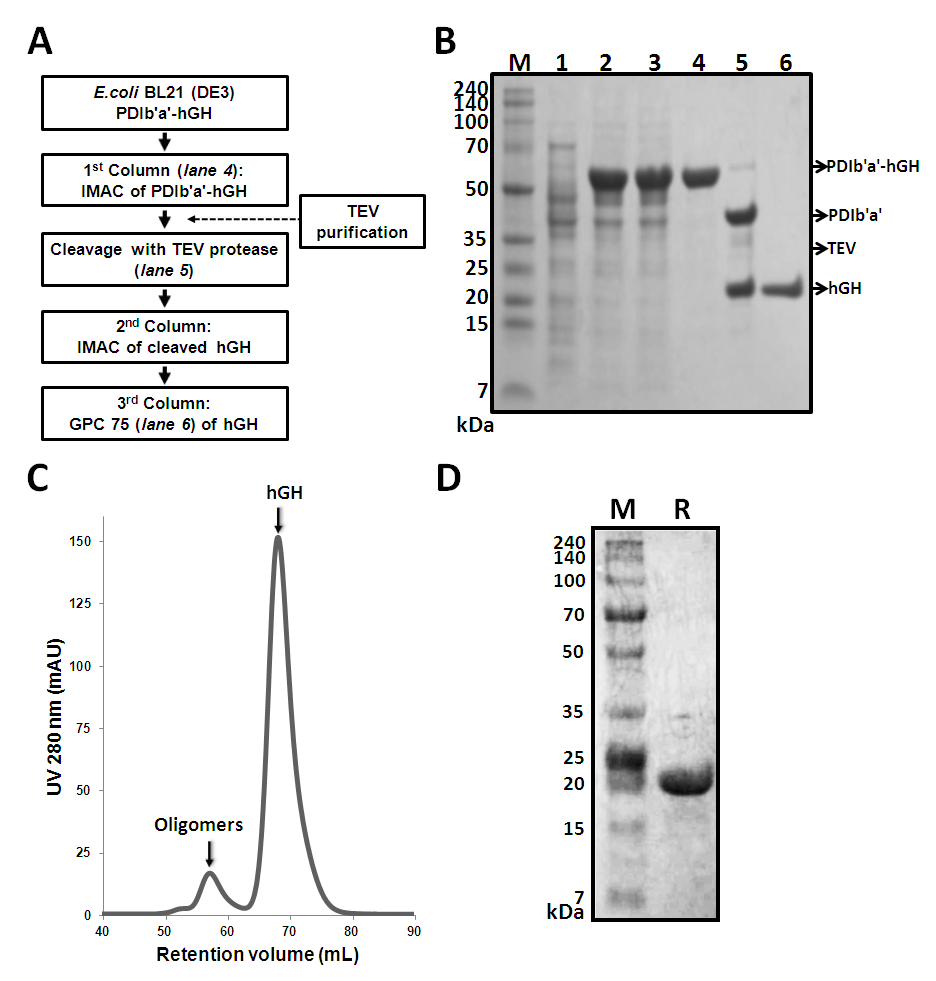


**Figure S2.**
